# Supplementary material for: Vaccine Efficacy of a Replication-Competent Interferon-Expressing Porcine Reproductive and Respiratory Syndrome (PRRS) Virus Against NADC-34 Challenge
Source: Vaccines (Basel). 2025 Apr 15;13(4):413. doi: 10.3390/vaccines13040413 (PMC12030877; doi:10.3390/vaccines13040413)
Supplement: Supplementary file 1 [file vaccines-13-00413-s001.zip › Supplementary File S2.pdf]

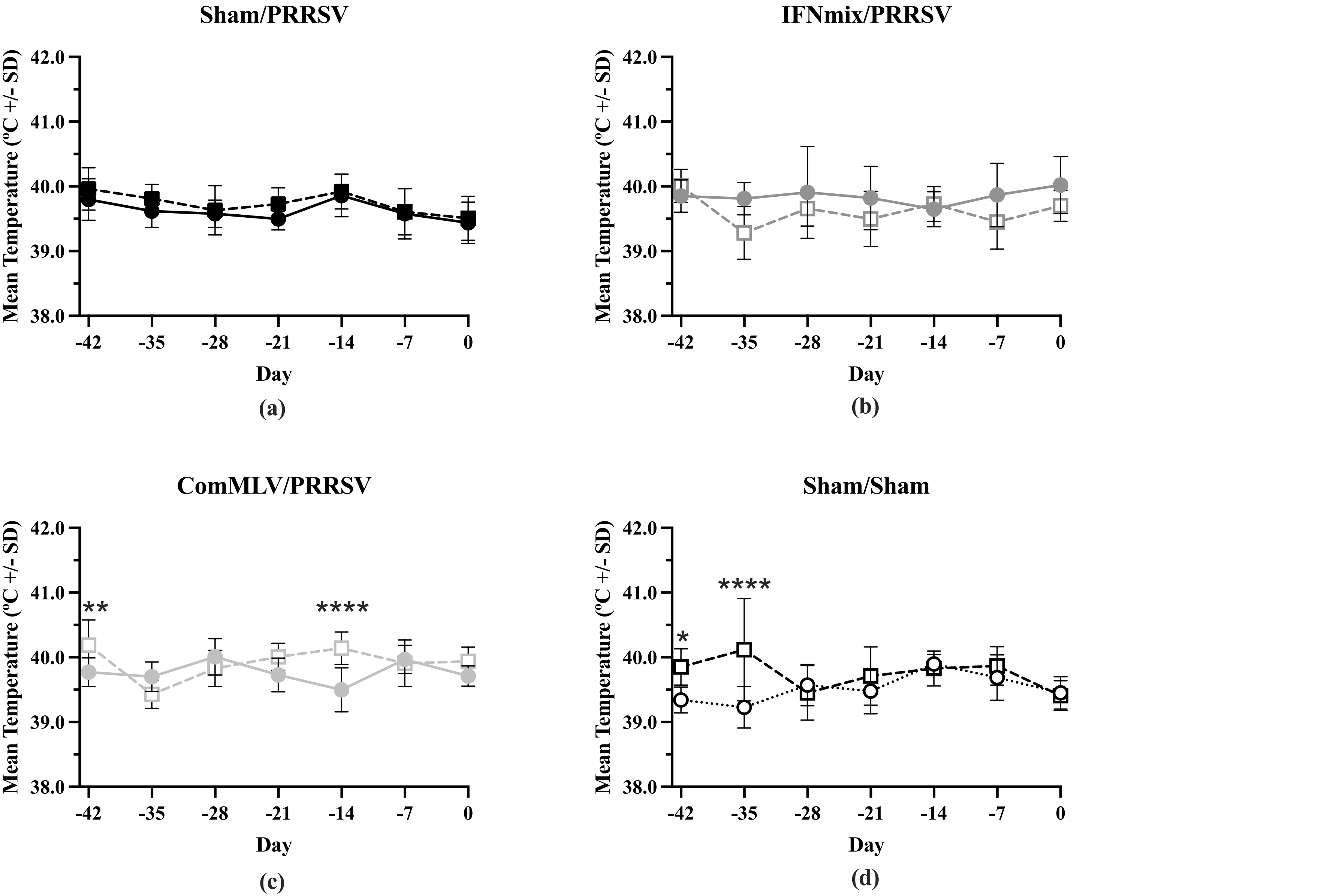

Figure S1. Pre-challenge temperatures of treatment groups in experiments 1 and 2. (a) Sham/PRRSV. Black circle, black line = experiment 1; Black square, dashed black line = experiment 2. **(b)** IFNmix/PRRSV. Dark gray circle, dark gray line = experiment 1; Dark gray open square, dark gray dashed line = experiment 2. **(c)** ComMLV/PRRSV. Light gray circle, light gray line = experiment 1; Light gray open square, light gray dashed line = experiment 2. **(d)** Sham/Sham. White circle, black dotted line = experiment 1; White filled square, black dashed line = experiment 2. * $p<0.05$, ** $p<0.01$, *** $p<0.001$, **** p=/<0.0001

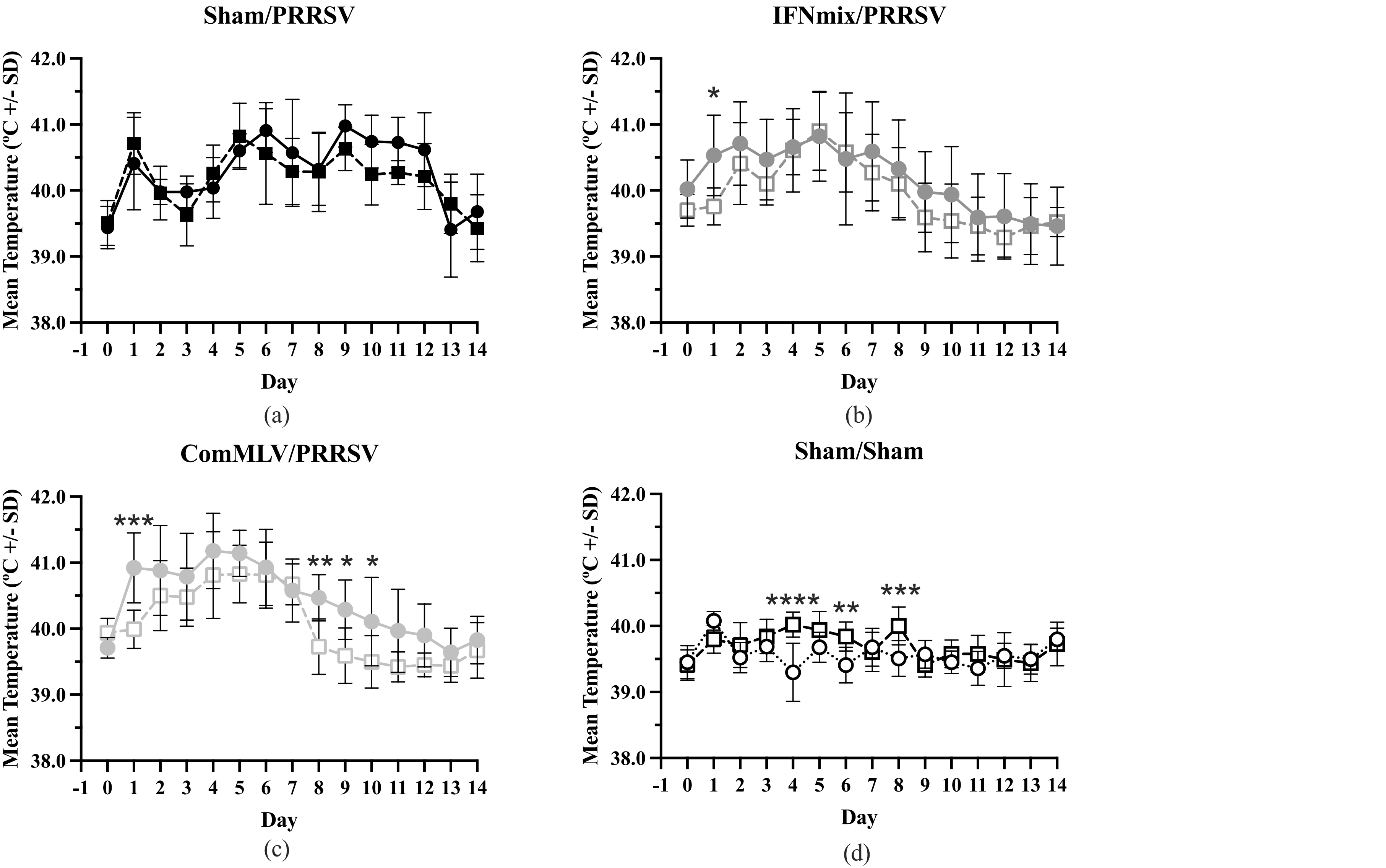

Figure S2. Post-challenge temperatures of treatment groups in experiments 1 and 2. (a) Sham/PRRSV. Black circle, black line = experiment 1; Black square, dashed black line = experiment 2. **(b)** IFNmix/PRRSV. Dark gray circle, dark gray line = experiment 1; Dark gray open square, dark gray dashed line = experiment 2. **(c)** ComMLV/PRRSV. Light gray circle, light gray line = experiment 1; Light gray open square, light gray dashed line = experiment 2. **(d)** Sham/Sham. White circle, black dotted line = experiment 1; White square, black dashed line = experiment 2.
* $p<0.05$, ** $p<0.01$, *** $p<0.001$, **** p=/<0.00011

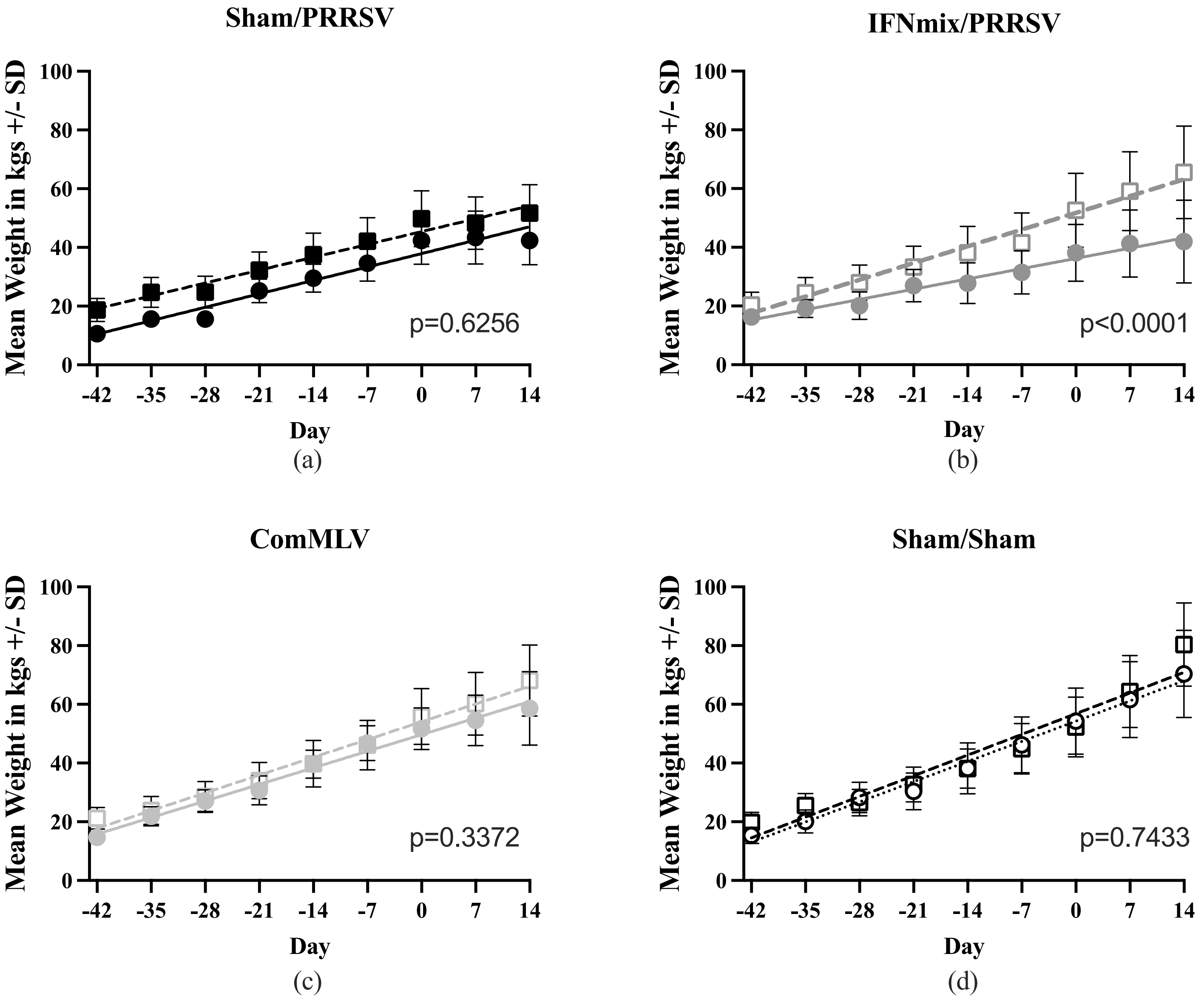

Figure S3. Simple linear regression lines for treatment groups in experiments 1 and 2. (a) Sham/PRRSV. Black circle, black line = experiment 1; Black square, black dashed line = experiment 2. **(b)** IFNmix/PRRSV. Dark gray circle, dark gray line = experiment 1; Dark gray open square, dark gray dashed line = experiment 2. **(c)** ComMLV/PRRSV. Light gray circle, light gray line = experiment 1; Light gray open square, light gray dashed line = experiment 2. **(d)** Sham/Sham. White circle, black dotted line = experiment 1; White square, black dashed line = experiment 2.

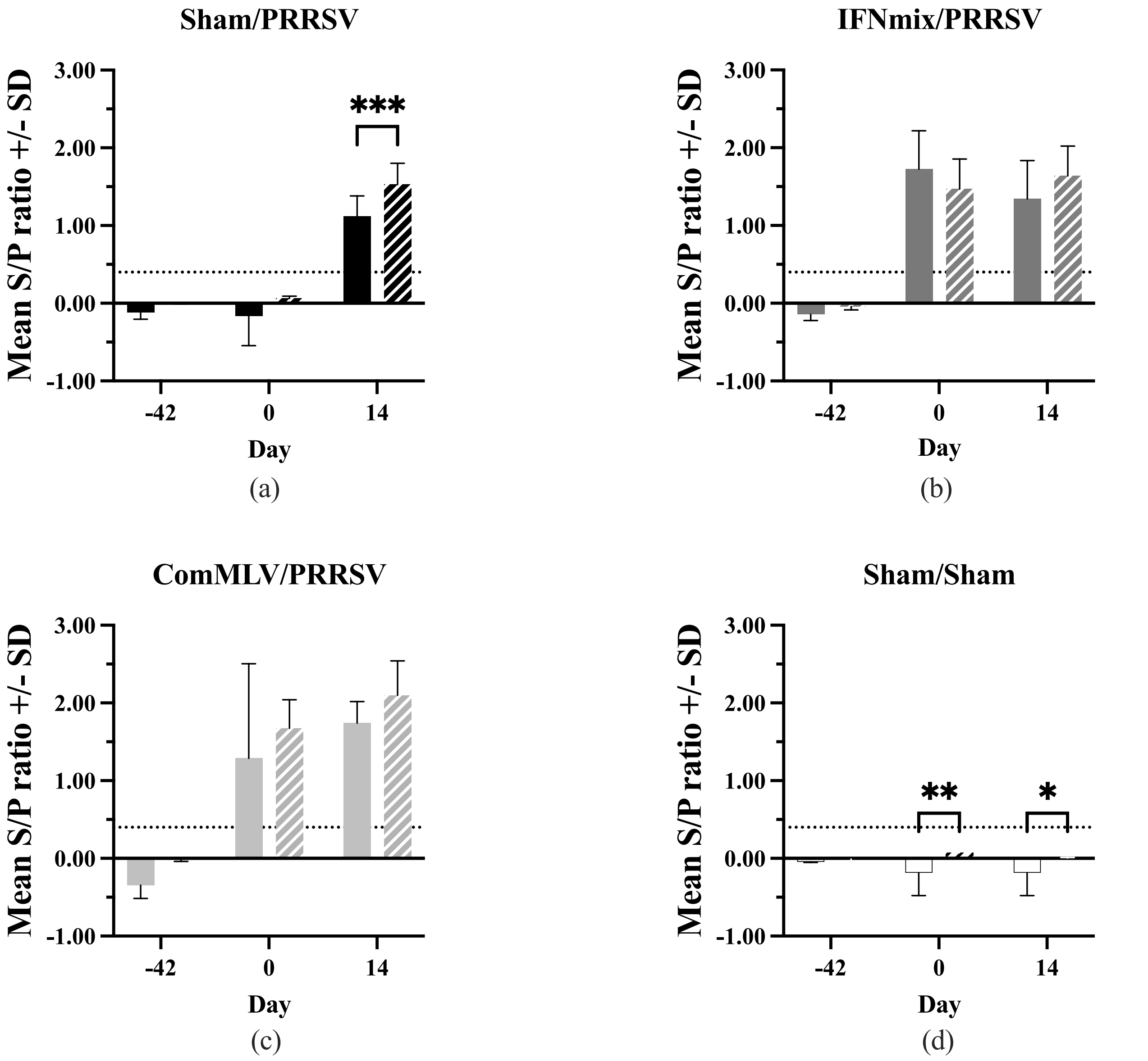

Figure S4. Mean S/P ratio of treatment groups in experiments 1 and 2. (a) Sham/PRRSV. Black = experiment 1; Black diagonal = experiment 2. **(b)** IFNmix/PRRSV. Dark gray = experiment 1; Dark gray diagonal = experiment 2. **(c)** ComMLV/PRRSV. Light gray = experiment 1; Light gray diagonal = experiment 2. **(d)** Sham/Sham. White = experiment 1;White diagonal = experiment 2. * $p<0.05$, **$p<0.01$, *** $p<0.001$, **** p=/<0.0001

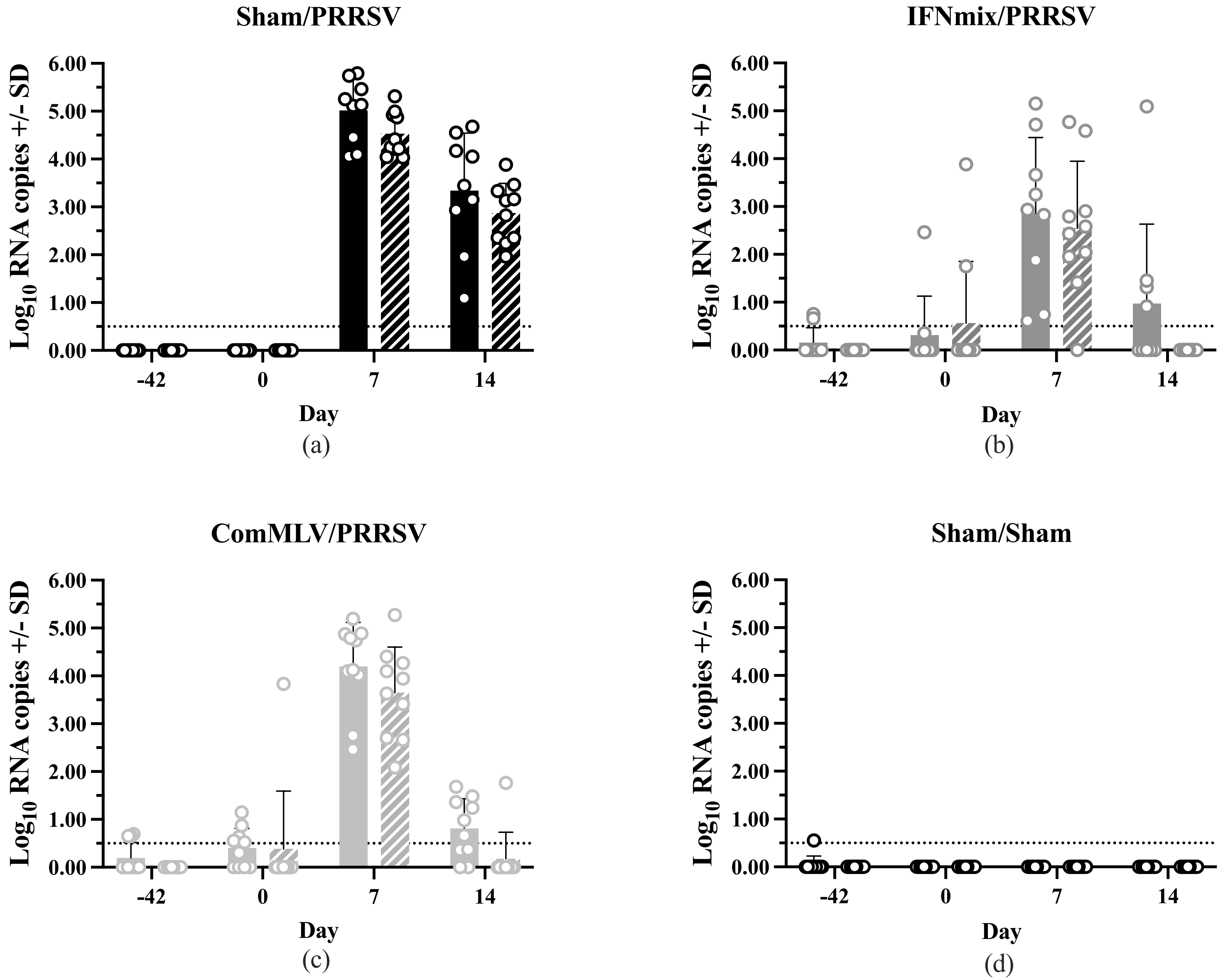

Figure S5. PRRSV RNA copies detected within treatment groups in experiments 1 and 2. Values shown are the mean $\log_{10}$ RNA copy number +/- standard deviation. Open circles represent the individual values within the treatment group. **(a)** Sham/PRRSV. Black = experiment 1; Black diagonal = experiment 2. **(b)** IFNmix/PRRSV. Dark gray = experiment 1; Dark gray diagonal = experiment 2. **(c)** ComMLV/PRRSV. Light gray = experiment 1; Light gray diagonal = experiment 2. **(d)** Sham/Sham. White = experiment 1; White diagonal = experiment 2. * p<0.05, **p<0.01, *** p<0.001, **** p=/<0.0001

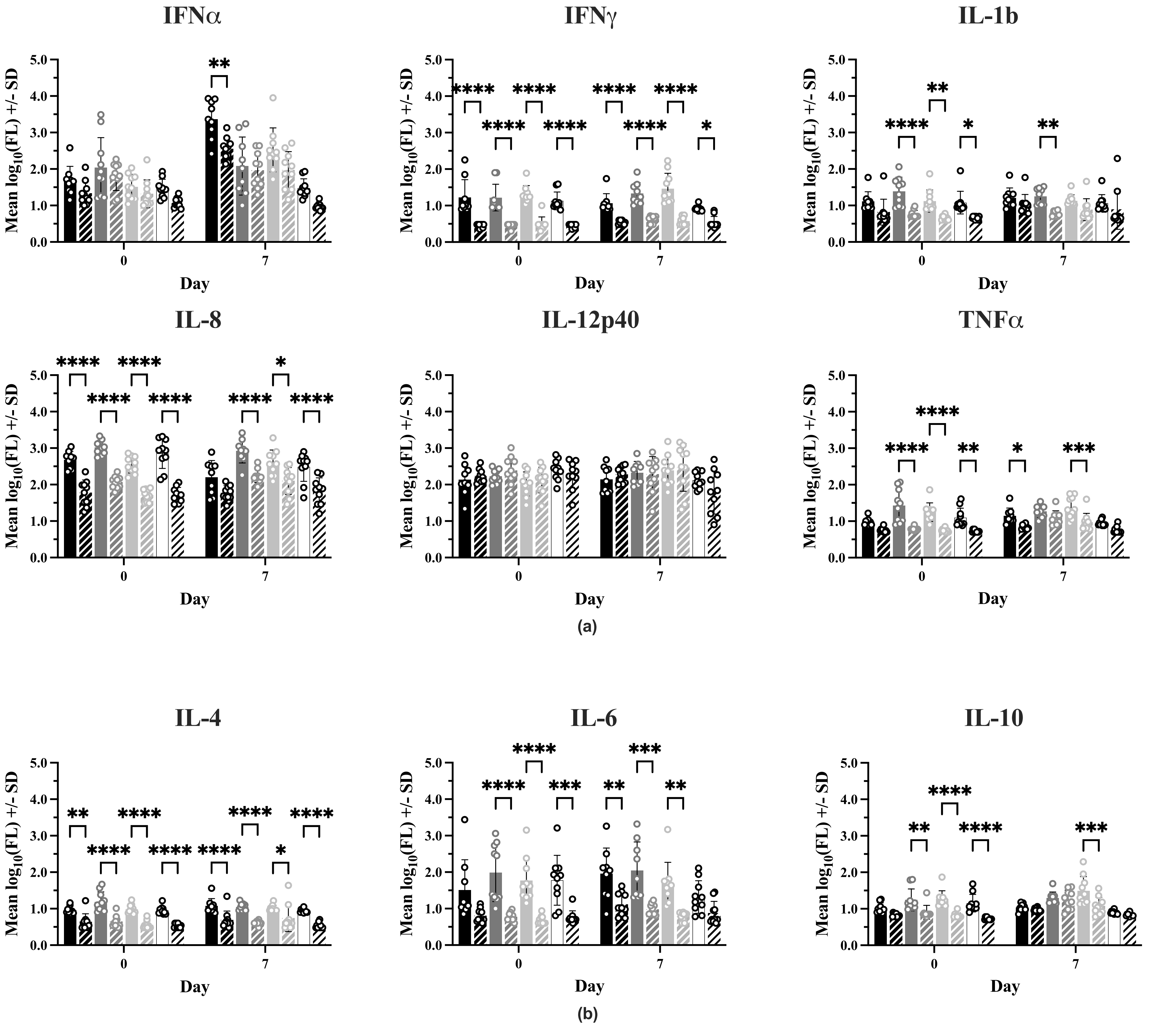

Figure S6. Cytokine concentrations by treatment group at Day 0 and Day 7 post-challenge in experiments 1 and 2. Values shown are the mean $\log_{10}$ median fluorescence +/- standard deviation. Open circles represent the individual values within treatment groups. Black = Sham/PRRSV experiment 1; Black diagonal = Sham/PRRSV experiment 2; Dark Gray = IFNmix/PRRSV experiment 1; Dark gray diagonal = IFNmix/PRRSV experiment 2; Light gray = ComMLV1/PRRSV experiment 1; Light gray diagonal = ComMLV2/PRRSV experiment 2; White = Sham/Sham experiment 1; White diagonal = Sham/Sham experiment 2. (a) Th1-associated cytokines. (b) Th2-associated cytokines. * p<0.05, **p<0.01, *** p<0.001, **** p=/<0.0001
